# Real-world use of enzalutamide in men with nonmetastatic castration-resistant prostate cancer in Japan

**DOI:** 10.1007/s10147-021-02070-z

**Published:** 2021-11-15

**Authors:** Akira Yokomizo, Junji Yonese, Shin Egawa, Hiroshi Fukuhara, Hiroji Uemura, Kazuo Nishimura, Masayoshi Nagata, Atsushi Saito, Takumi Lee, Susumu Yamaguchi, Norio Nonomura

**Affiliations:** 1 Department of Urology, Harasanshin Hospital, 1-8 Taihakumachi, Hakata-ku, Fukuoka, 812-0033 Japan; 2 Department of Genitourinary Oncology, Cancer Institute Hospital of Japanese Foundation for Cancer Research, 3-8-31 Ariake, Koto-ku, Tokyo, 135-8550 Japan; 3 Department of Urology, Jikei University School of Medicine, 3-25-8 Nishishimbashi, Minato-ku, Tokyo, 105-8461 Japan; 4 Department of Urology, Kyorin University School of Medicine, 6-20-2, Mitaka, Tokyo, 181-8611 Japan; 5 Department of Urology and Renal Transplantation, Yokohama City University Medical Center, 4-57 Urafune-cho, Minami-ku, Yokohama, 232-0024 Japan; 6 Department of Urology, Osaka International Cancer Institute, 3-1-69 Otemae, Chuo-ku, Osaka, 541-8567 Japan; 7 Department of Urology, Juntendo University Graduate School of Medicine, 2-1-1 Hongo, Bunkyo-ku, Tokyo, 113-8421 Japan; 8 Medical Affairs Japan, Astellas Pharma Inc., 2-5-1 Nihonbashi-Honcho, Chuo-ku, Tokyo, 103-8411 Japan; 9 Development, Astellas Pharma Inc., 2-5-1 Nihonbashi-Honcho, Chuo-ku, Tokyo, 103-8411 Japan; 10 Department of Urology, Osaka University Graduate School of Medicine, 2-2 Yamadaoka, Suita, 565-0871 Japan

**Keywords:** Efficacy, Enzalutamide, Japan, Nonmetastatic castration-resistant prostate cancer, Real world, Safety

## Abstract

**Background:**

The purpose of the study is to evaluate real-world effectiveness and safety of enzalutamide in men with nonmetastatic castration-resistant prostate cancer (nmCRPC) in Japan.

**Methods:**

This was a retrospective evaluation of medical records from men in Japan who started enzalutamide treatment from November 1, 2014, to March 31, 2018, and received androgen deprivation therapy throughout. The primary endpoint was time to prostate-specific antigen (PSA) progression. Secondary endpoints included PSA response rate, time to first use of new antineoplastic therapy, time to first use of cytotoxic chemotherapy, and enzalutamide treatment duration. An exploratory analysis of metastasis-free survival (MFS) was also performed. Adverse events (AEs) were analyzed to assess safety.

**Results:**

Based on data from medical records of 205 men in Japan, median time to PSA progression was 27 months (95% confidence interval [CI] 19–not reached [NR]), with 82.5% and 52.0% of men achieving PSA response rates of ≥ 50% and ≥ 90%, respectively. Median time to first use of new antineoplastic therapy was 36 months (95% CI 27−NR) and median enzalutamide treatment duration was 13 months (interquartile range: 7–24). Median time to first use of cytotoxic chemotherapy was NR (95% CI 41−NR). Median MFS was 29 months (95% CI 23−35). In total, 51.7% of men experienced AEs, with malaise (18.5%), decreased appetite (10.7%), and nausea (4.9%) the most frequently reported.

**Conclusions:**

This is the first study to demonstrate the real-world effectiveness and safety of enzalutamide in men with nmCRPC in Japan, further informing healthcare providers about available treatment options for this patient population.

**Supplementary Information:**

The online version contains supplementary material available at 10.1007/s10147-021-02070-z.

## Introduction

The incidence of prostate cancer has been increasing worldwide in recent years. In Asian countries, the steady increase in prostate cancer incidence is thought to be associated with a number of factors, including increased prostate-specific antigen (PSA) testing, the development of cancer registries, an aging population, and environmental factors such as diet and obesity [1]. In 2018, the World Health Organization’s GLOBOCAN project revealed a worldwide prostate cancer incidence of 1,276,106. By region, the highest incidence was reported in Europe (449,761), followed by Asia (297,215) and North America (234,278) [2]. The incidence of prostate cancer in Japan was estimated to be 78,400, with prostate cancer sixth in cancer-related mortality in men, resulting in an estimated 12,400 deaths [3].

Enzalutamide has been approved by Japan’s Ministry of Health, Labour and Welfare for the treatment of men with castration-resistant prostate cancer (CRPC) since 2014 [4], based on multiple studies [5–7]. In 2020, it was also approved in men with prostate cancer with distant metastases [4], based on the ARCHES [8] and ENZAMET [9] trials. Despite enzalutamide use in clinical practice for the treatment of patients with either metastatic or nonmetastatic (nm) CRPC, there are currently no prospective or retrospective studies on the use of enzalutamide in men with nmCRPC in Japan. The PROSPER study demonstrated the efficacy and safety of enzalutamide in men with nmCRPC; however, Japanese men were not included in this study population [10]. Recently, both apalutamide and darolutamide have been approved specifically for the treatment of men with nmCRPC in Japan [11–14].

To further inform healthcare providers of the treatment options for men with nmCRPC in Japan, we evaluated the real-world effectiveness and safety of enzalutamide in this patient population.

## Patients and methods

### Study design

This retrospective evaluation of medical records assessed the real-world effectiveness and safety of enzalutamide in men with nmCRPC in Japan. The observation period for effectiveness and safety data was from the index date (date of first prescription of enzalutamide from November 1, 2014, to March 31, 2018) to the last visit; however, clinical data collection was performed from medical records dated up to September 30, 2018. Data extraction was performed from October 1, 2018, to January 31, 2019, whereby investigators completed a case report form for eligible patients, based on their medical records, which was then entered into the database at EP-CRSU Co., Ltd. Data analysis was completed on June 30, 2019.

### Study sites and patients

Study sites were selected based on their experience of treating patients with enzalutamide. The study protocol was reviewed and approved by the ethics committee at each study site.

Men aged ≥ 20 years on the index date who started treatment with enzalutamide from November 1, 2014, to March 31, 2018, and received androgen deprivation therapy (ADT) throughout the treatment period were eligible for inclusion. Patients were required to have PSA progression despite ADT prior to enzalutamide treatment, at least two PSA measurements before the index date (the first obtained within 3 months before the index date and the second obtained at least 3 weeks prior to the first) and at least one PSA measurement after the index date, and confirmed absence of distant metastases via bone scintigraphy, computed tomography, or magnetic resonance imagery within 6 months prior to the initiation of enzalutamide therapy, and to provide written informed consent for the use of data for the study prior to the initiation of data extraction. Men with a history of distant metastases or those who had received abiraterone, enzalutamide, docetaxel, cabazitaxel, or another investigational product for the treatment of prostate cancer prior to the index date were excluded.

To avoid screening bias towards men with a favorable response to enzalutamide, all men who met the inclusion criteria were enrolled in the study, including those who died during the observation period.

### Endpoints

The primary endpoint was time from the index date to unconfirmed PSA progression during treatment with enzalutamide, selected due to the regularity of PSA assessment during routine clinical practice in Japan. PSA progression was defined as a ≥ 25% and ≥ 2 ng/mL increase from the PSA nadir in men who had a reduction in PSA after the index date. In men with no reduction in PSA after the index date, PSA progression was defined as a ≥ 25% and ≥ 2 ng/mL increase from the PSA value on the index date or immediately before the index date (baseline). Data were censored on the date of the last PSA measurement during enzalutamide treatment or on the index date in men with no PSA measurements during enzalutamide treatment.

Secondary endpoints included PSA response rate, time to first use of new antineoplastic therapy, time to first use of cytotoxic chemotherapy, and treatment duration of enzalutamide. PSA response rate was defined as the proportion of men achieving a ≥ 50% or ≥ 90% decline in the lowest PSA from baseline during treatment with enzalutamide; men without any PSA measurements during treatment with enzalutamide were not included in this analysis. Time to first use of new antineoplastic therapy was defined as the time from index date to initiation of chemotherapy, hormone therapy, or radiopharmaceutical for prostate cancer after enzalutamide treatment. Data were censored on the date of the last visit during the observation period. Time to first use of cytotoxic chemotherapy was defined as the time from index date to initiation of cytotoxic therapy for prostate cancer. Again, data were censored on the date of the last visit during the observation period. Treatment duration of enzalutamide was defined as the time from index date to discontinuation of treatment for > 60 consecutive days or the end of the observation period.

An exploratory analysis of metastasis-free survival (MFS) was also performed to enable comparison of results with those from published clinical trials. MFS was defined as the time from index date to confirmed metastasis via imaging or death. Data were censored on the date of the last confirmation of nonmetastasis via imaging during the observation period, or on the index date for men without any imaging during enzalutamide treatment.

In addition, subgroup analyses were performed by PSA doubling time (≤ 10 months/> 10 months), age (< 75 years/≥ 75 years), Gleason score (< 8/≥ 8), and median PSA at index date (< median/≥ median). PSA doubling time was calculated using the Pound et al. method [15]. Post hoc subgroup analyses by initial daily dose of enzalutamide (40–120 mg/160 mg) were also performed. Results of the primary endpoint analyses are presented, as well as MFS, by median PSA at index date.

Adverse events (AEs) were analyzed to assess the safety of enzalutamide. Additionally, patient background information and data related to prostate cancer treatment history were collected.

### Statistical analyses

Statistical analyses were performed using SAS, version 9.4 (SAS Institute, Cary, NC, USA). Data for continuous variables were summarized using descriptive statistics. The Kaplan–Meier method was used to estimate the median and 95% confidence intervals (CIs). Categorical variables were analyzed using frequency and proportion, with missing categorical variables handled as “unknown.” Subgroup analyses were performed using the Cox proportional hazards model. No adjustment was made for multiplicity. Statistical inference in relation to all subgroup analyses should be considered exploratory; therefore, these results should be interpreted with caution.

## Results

### Patient characteristics

Data were extracted from the medical records of 216 men across 28 sites in Japan. Overall, 205 men met all inclusion criteria and were included in the analysis set; 11 men were excluded due to not starting enzalutamide treatment from November 1, 2014, to March 31, 2018, (*n* = 7) or due to the absence of distant metastases not being confirmed via bone scintigraphy, computed tomography, or magnetic resonance imagery within 6 months of enzalutamide therapy initiation (*n* = 4).

Patient background and treatment history are presented in Table 1. Gleason score was ≥ 8 at diagnosis in 60.0% of men. Median PSA at baseline was 6.3 ng/mL and PSA doubling time was ≤ 10 months in 87.8% of men. In total, 43.4% of men did not receive prior curative therapy.

**Table 1:** Patient background and treatment history

Characteristics	Analysis set (*n* = 205)
Age at index date, years	
Mean ± SD	76.2 ± 7.2
< 75, *n* (%)	84 (41.0)
≥ 75, *n* (%)	121 (59.0)
Baseline body weight, kg, mean ± SD^a^	62.3 ± 10.1
Baseline BMI, mean ± SD^b^	23.2 ± 3.2
Gleason score at prostate cancer diagnosis, *n* (%)	
< 8	59 (28.8)
≥ 8	123 (60.0)
Unknown	23 (11.2)
Baseline PSA, ng/mL^c^	
Mean ± SD	19.6 ± 93.1
Median (range)	6.3 (0–1306.1)
Baseline PSA doubling time, months	
Mean ± SD	6 ± 21
≤ 10, *n* (%)	180 (87.8)
> 10, *n* (%)	21 (10.2)
Not measurable, *n* (%)	4 (2.0)
Comorbidities (except prostate cancer) at initiation of enzalutamide, *n* (%)	
No	44 (21.5)
Yes	140 (68.3)
Unknown	21 (10.2)
Prior curative therapy, *n* (%)	
Yes	115 (56.1)
Surgery	42 (20.5)
Radiotherapy	57 (27.8)
Both	16 (7.8)
No	89 (43.4)
Unknown	1 (0.5)
Median (range) time from diagnosis of prostate cancer to initiation of enzalutamide, months^d^	70 (4–244)
Median (range) time from initiation of ADT to initiation of enzalutamide, months^e^	56 (3–227)
Initial daily dose of enzalutamide, *n* (%)^f^	
40–80 mg	24 (11.7)
120 mg	25 (12.2)
160 mg	156 (76.1)

*ADT* androgen deprivation therapy; *BMI* body mass index; *PSA* prostate-specific antigen; *SD* standard deviation

^a^Unknown: *n* = 44 (21.5%)

^b^Unknown: *n* = 47 (22.9%)

^c^Baseline PSA defined as PSA measured within 3 weeks prior to the index date

^d^Unknown: *n* = 10 (4.9%)

^e^Unknown: *n* = 7 (3.4%)

^f^The standard daily dose of enzalutamide is 160 mg; however, in this study, data were collected regardless of actual dose

The majority of men (76.1%) received an initial daily dose of enzalutamide of 160 mg (Table 1). From the initial daily dose, an increase was reported in 14 men (6.8%) and a reduction was reported in 56 men (27.3%). The most common reasons for enzalutamide treatment discontinuation were disease progression (54.6%), AEs (28.7%), patient decision (11.1%), loss to follow-up (1.9%), and other (11.1%).

### Primary endpoint

**Fig. 1:**
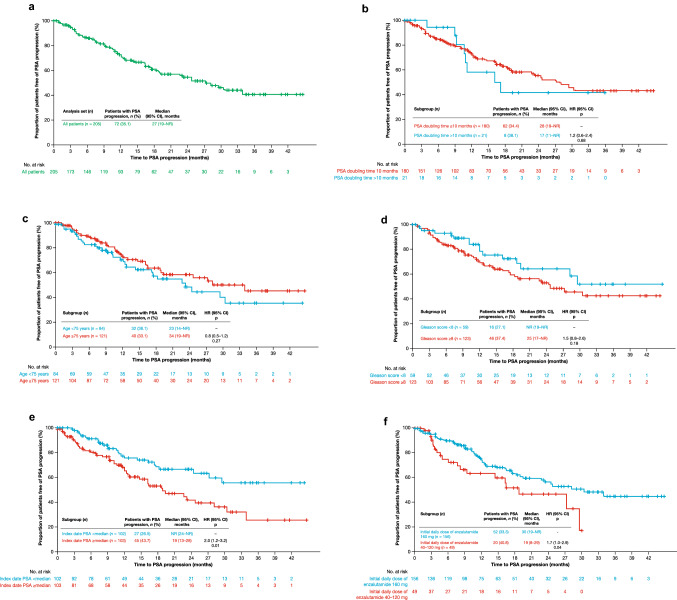
Time to PSA progression (**a**) in all patients and in subgroup analyses by (**b**) PSA doubling time, **c** age, **d** Gleason score, **e** median PSA at index date, and (**f**) initial daily dose of enzalutamide. *CI* confidence interval; *HR* hazard ratio; *NR* not reached; *PSA* prostate-specific antigen

Overall, 72 (35.1%) men experienced PSA progression. Median time to PSA progression was 27 months (95% CI 19−not reached [NR]) [Table 2; Fig. 1a].

**Table 2:** Effectiveness analyses

Endpoint	Result
Primary endpoint	
Median (95% CI) time to PSA progression, months	27 (19−NR)
Secondary endpoints	
PSA response rate,^a^ *n* (%) [95% CI]	
≥ 50% decrease	165 (82.5) [76.5−87.5]
≥ 90% decrease	104 (52.0) [44.8−59.1]
Median (95% CI) time to first use of new antineoplastic therapy, months	36 (27−NR)
Median (95% CI) time to first use of cytotoxic chemotherapy, months	NR (41−NR)
Median (IQR) enzalutamide treatment duration, months	13 (7–24)
Exploratory endpoint	
Median (95% CI) metastasis-free survival, months	29 (23–35)

Analysis set: *n* = 205

*CI* confidence interval; *IQR* interquartile range; *NR* not reached; *PSA* prostate-specific antigen

^a^*n* = 200 men with PSA measurements during the treatment period

### Secondary endpoints

**Fig. 2:**
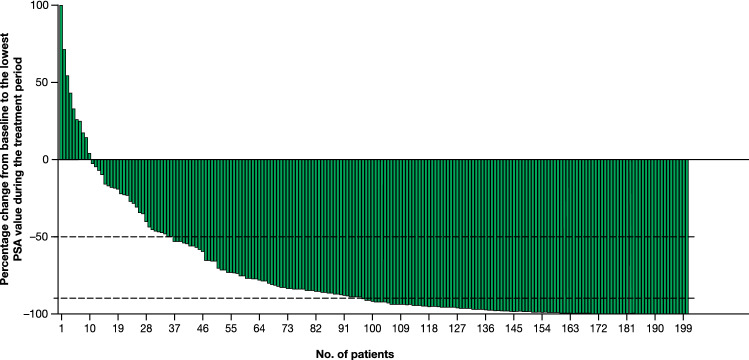
≥ 50% and ≥ 90% PSA response rates. *n* = 200 men with PSA measurements during the treatment period. Data presented in descending order of percentage change. *PSA* prostate-specific antigen

Among the 200 (97.6%) men who had PSA measurements during the treatment period, 165 (82.5%) achieved a ≥ 50% decrease in PSA (95% CI 76.5−87.5) and 104 (52.0%) achieved a ≥ 90% decrease in PSA (95% CI 44.8−59.1) [Table 2]. PSA response rates in descending order of percentage change from baseline to the lowest PSA observed during the treatment period are presented in Fig. 2.

Median time to first use of new antineoplastic therapy was 36 months (95% CI 27–NR) [Table 2; Online Resource Fig. 1]. In total, 70 (34.1%) men initiated a new antineoplastic therapy; details of the new antineoplastic therapies initiated are provided in Online Resource Table S1.

Median time to first use of cytotoxic chemotherapy was NR (95% CI 41–NR) [Table 2; Online Resource Fig. 2]. Only 41 (20.0%) men initiated cytotoxic chemotherapy, 31 of whom used docetaxel (Online Resource Table 2).

Median enzalutamide treatment duration was 13 months (interquartile range: 7–24) [Table 2]. The largest number of men (*n* = 52; 25.4%) had a treatment duration of > 6 months to ≤ 1 year and 15 (7.3%) men received enzalutamide treatment for 3 years or longer (Online Resource Table 3).

### Exploratory endpoint

Overall, 59 men had an MFS event and median MFS was 29 months (95% CI 23–35) [Table 2; Online Resource Fig. 3a].

### Subgroup analyses

#### Primary endpoint

Subgroup analyses revealed that there was no significant difference in time to PSA progression between men with PSA doubling time ≤ 10 months and > 10 months (Fig. 1b), men aged < 75 years and ≥ 75 years (Fig. 1c), and men with a Gleason score < 8 and ≥ 8 (Fig. 1d). Time to PSA progression was significantly longer for men with index date PSA below the median (6.3 ng/mL) versus those with index date PSA equal to or above the median (*p* = 0.01) [Fig. 1e], and for those who received an initial daily dose of enzalutamide of 160 mg versus those who received an initial daily dose of 40–120 mg (*p* = 0.04) [Fig. 1f].

#### Exploratory endpoint

Subgroup analysis of MFS by median PSA at index date revealed that there was no significant difference between men with index date PSA below the median (6.3 ng/mL) and those with index date PSA equal to or above the median (Online Resource Fig. 3b).

### Safety

A total of 108 (52.7%) men discontinued enzalutamide prior to the final day of observation, with worsening of condition (*n* = 59) and AEs (*n* = 31) the most frequently reported reasons for discontinuation.

In total, 106 (51.7%) men experienced AEs during the observation period. The most frequently reported AEs, by preferred term, were malaise (*n* = 38; 18.5%), decreased appetite (*n* = 22; 10.7%), and nausea (*n* = 10; 4.9%) [Table 3].

**Table 3:** AEs occurring in ≥ 2% of men during the observation period

Preferred term	Men, *n* (%)
Malaise	38 (18.5)
Decreased appetite	22 (10.7)
Nausea	10 (4.9)
Fatigue	9 (4.4)
Hot flush	7 (3.4)
Dysgeusia	6 (2.9)
Constipation	4 (2.0)
Blood lactate dehydrogenase increased	4 (2.0)
Hypertension	4 (2.0)
Nasopharyngitis	4 (2.0)

Analysis set: *n* = 205

Medical Dictionary for Regulatory Activities, version 22.0

*AE* adverse event

Adverse drug reactions were reported in 103 (50.2%) men, the incidence of which was slightly higher in those receiving 160 mg of enzalutamide (*n* = 85; 54.5%) compared to 40–120 mg of enzalutamide (*n* = 18; 36.7%). Severe adverse drug reactions were experienced by 14 (6.8%) men, with only malaise and nausea occurring in ≥ 3 men or at an incidence of > 1%.

## Discussion

This retrospective evaluation of 205 men with nmCRPC in Japan who were receiving treatment with enzalutamide revealed that median time to PSA progression was 27 months (95% CI 19−NR), with 82.5% and 52.0% of men achieving PSA response rates of ≥ 50% and ≥ 90%, respectively. Median time to first use of new antineoplastic therapy was 36 months (95% CI 27−NR) and median enzalutamide treatment duration was 13 months (interquartile range: 7–24). Exploratory analysis revealed that median MFS was 29 months (95% CI 23−35). Subgroup analyses revealed that time to PSA progression was significantly longer in men with index date PSA below the median, suggesting that elevated PSA increases the likelihood of progression, and in men who received an initial daily dose of enzalutamide of 160 mg, suggesting that a lower dose of enzalutamide may confer reduced efficacy. In total, 51.7% of men experienced AEs; malaise (18.5%), decreased appetite (10.7%), and nausea (4.9%) were most frequently reported.

Our study provides valuable insights into the effectiveness and safety of enzalutamide in the real-world treatment of men with nmCRPC in Japan. These data are highly relevant to day-to-day clinical practice, with time to PSA progression selected as the primary endpoint and other PSA-related variables analyzed as secondary endpoints to align with the regularity of PSA assessment during routine treatment monitoring in Japan.

In the PROSPER randomized controlled trial (ClinicalTrials. gov NCT02003924) [10, 16], which included 1401 men with nmCRPC, median overall survival was significantly increased with enzalutamide versus placebo (hazard ratio: 0.73; 95% CI 0.61–0.89; *p* = 0.001). Median MFS in men receiving enzalutamide (160 mg) plus ADT in PROSPER was 37 months, slightly longer than the 29 months observed in our exploratory analysis. Time to PSA progression was 37 months and time to first antineoplastic therapy was 40 months, both of which are also slightly longer than the corresponding findings from our trial (27 months and 36 months, respectively). It should be considered that in routine clinical practice in Japan, when PSA levels are maintained, radiographic imaging is generally not performed. As a result, there were 146 censored patients during analysis of MFS in our study, limiting the reproducibility of findings between our retrospective analysis of medical records and those observed in a controlled clinical trial environment. The incidence of AEs in men receiving enzalutamide in PROSPER was slightly lower than in our analysis (31% versus 52%, respectively), with fatigue the most commonly reported AE, occurring in 33% of men receiving enzalutamide. These differences observed between a selected patient group and our real-world population are unsurprising, given that our population is slightly older and likely to have more comorbidities, in addition to therapeutic dose differences. It will be helpful to consider our findings in line with results from the JCASTRE-Zero (ClinicalTrials.gov NCT02588001) observational study of enzalutamide in men with nmCRPC occurring after curative therapy, once published.

Our analyses have some inherent limitations that should be considered. The lack of a control group for comparison limits the interpretation of the effectiveness and safety of enzalutamide. Due to the retrospective, observational nature of this study, the collectable information was limited to the data included in patients’ medical records. There is also potential for selection bias at study sites and disproportionate capture of men being treated at larger medical institutions, where imaging tests are more likely to be available in a timely manner. Therefore, caution must be exercised if the results of our study are to be generalized for the treatment of nmCRPC. In addition, results from the subgroup analyses must be interpreted with caution given that the study was not designed or powered for such analyses and due to small patient numbers in some groups.

While enzalutamide is approved for the treatment of CRPC, both apalutamide and darolutamide have recently been approved specifically for nmCRPC in Japan. In the SPARTAN trial (ClinicalTrials.gov NCT01946204) in 1207 men with nmCRPC [11], median MFS was 41 months following apalutamide and in the ARAMIS trial (ClinicalTrials.gov NCT02200614) in 1509 men with nmCRPC [12], median MFS was 40 months with darolutamide. While this is approximately 11 months longer than the results observed with enzalutamide in our real-world analysis, the efficacy of apalutamide and darolutamide in clinical practice has not been reported. The results of our subgroup analyses indicating that time to PSA progression was significantly longer in men with index date PSA < median (6.3 ng/mL) are consistent with findings from an analysis of the SPARTAN study in which PSA > median (5.5 ng/dL) was found to be a significant predictor of metastatic disease [17]. Similarly, no significant difference in time to PSA progression was observed between men with a PSA doubling time ≤ 10 months versus > 10 months, men aged < 75 years versus ≥ 75 years, and men with a Gleason score < 8 versus ≥ 8 in our study, consistent with findings that shorter PSA doubling time (≤ 6 months), older age (≥ 65 years), and higher Gleason score (≥ 8) were not significant predictors of metastatic disease for patients enrolled in SPARTAN [17]. Enzalutamide has been widely used in clinical practice since its approval in 2014; thus, physicians in Japan have a good understanding of the efficacy and safety of enzalutamide for the treatment of nmCRPC compared to other, more recently available treatment options.

## Conclusion

This real-world evidence, retrospective study has evaluated the effectiveness and safety of enzalutamide in men with nmCRPC in Japan in clinical practice. These data, along with data from additional real-world analyses, are beneficial to further inform healthcare providers about available treatment options in men with nmCRPC in Japan.

## Supplementary Information

Below is the link to the electronic supplementary material.

Supplementary file1 (PDF 421 KB)

## Data Availability

Researchers may request access to anonymized participant-level data, trial-level data, and protocols from Astellas-sponsored clinical trials at www.clinicalstudydatarequest.com. For the Astellas criteria on data sharing see: https://clinicalstudydatarequest.com/Study-Sponsors/Study-Sponsors-Astellas.aspx.
