# Correction to: Real-world use of enzalutamide in men with nonmetastatic castration-resistant prostate cancer in Japan

**DOI:** 10.1007/s10147-022-02126-8

**Published:** 2022-02-01

**Authors:** Akira Yokomizo, Junji Yonese, Shin Egawa, Hiroshi Fukuhara, Hiroji Uemura, Kazuo Nishimura, Masayoshi Nagata, Atsushi Saito, Takumi Lee, Susumu Yamaguchi, Norio Nonomura

**Affiliations:** 1 Department of Urology, Harasanshin Hospital, 1-8 Taihakumachi, Hakata-ku, Fukuoka, 812-0033 Japan; 2 Department of Genitourinary Oncology, Cancer Institute Hospital of Japanese Foundation for Cancer Research, 3-8-31 Ariake, Koto-ku, Tokyo, 135-8550 Japan; 3 Department of Urology, Jikei University School of Medicine, 3-25-8 Nishishimbashi, Minato-ku, Tokyo, 105-8461 Japan; 4 Department of Urology, Kyorin University School of Medicine, 6-20-2, Mitaka, Tokyo, 181-8611 Japan; 5 Department of Urology and Renal Transplantation, Yokohama City University Medical Center, 4-57 Urafune-cho, Minami-ku, Yokohama, 232-0024 Japan; 6 Department of Urology, Osaka International Cancer Institute, 3-1-69 Otemae, Chuo-ku, Osaka, 541-8567 Japan; 7 Department of Urology, Juntendo University Graduate School of Medicine, 2-1-1 Hongo, Bunkyo-ku, Tokyo, 113-8421 Japan; 8 Medical Affairs Japan, Astellas Pharma Inc., 2-5-1 Nihonbashi-Honcho, Chuo-ku, Tokyo, 103-8411 Japan; 9 Development, Astellas Pharma Inc., 2-5-1 Nihonbashi-Honcho, Chuo-ku, Tokyo, 103-8411 Japan; 10 Department of Urology, Osaka University Graduate School of Medicine, 2-2 Yamadaoka, Suita, 565-0871 Japan

## Correction to: International Journal of Clinical Oncology 10.1007/s10147-021-02070-z

The article, Real‑world use of enzalutamide in men with nonmetastatic castration‑resistant prostate cancer in Japan written by Akira Yokomizo, Junji Yonese, Shin Egawa, Hiroshi Fukuhara, Hiroji Uemura, Kazuo Nishimura, Masayoshi Nagata, Atsushi Saito, Takumi Lee, Susumu Yamaguchi, and Norio Nonomura was originally published Online First without Open Access. With the author(s)’ decision to opt for Open Choice the copyright of the article changed on January 31, 2022 to © Author(s) 2021 and the article is forthwith distributed under a Creative Commons Attribution 4.0 International License, which permits use, sharing, adaptation, distribution and reproduction in any medium or format, as long as you give appropriate credit to the original author(s) and the source, provide a link to the Creative Commons licence, and indicate if changes were made. The images or other third party material in this article are included in the article’s Creative Commons licence, unless indicated otherwise in a credit line to the material. If material is not included in the article’s Creative Commons licence and your intended use is not permitted by statutory regulation or exceeds the permitted use, you will need to obtain permission directly from the copyright holder. To view a copy of this licence, visit http://creativecommons.org/licenses/by/4.0.

The original article has been corrected.

