# Supplementary material for: Real-world use of enzalutamide in men with nonmetastatic castration-resistant prostate cancer in Japan
Source: Int J Clin Oncol. 2021 Nov 15;27(2):418–26. doi: 10.1007/s10147-021-02070-z (PMC8816761; doi:10.1007/s10147-021-02070-z)
Supplement: Supplementary file 1 — Supplementary file1 (PDF 421 KB) [file 10147_2021_2070_MOESM1_ESM.pdf]

Supplementary material

Real-world use of enzalutamide in men with nonmetastatic castration-resistant prostate cancer in Japan

Int J Clin Oncol

Akira Yokomizo* • Junji Yonese • Shin Egawa • Hiroshi Fukuhara • Hiroji Uemura • Kazuo Nishimura • Masayoshi Nagata • Atsushi Saito • Takumi Lee • Susumu Yamaguchi • Norio Nonomura

* **Corresponding author** Director, Department of Urology, Harasanshin Hospital, 1-8 Taihakumachi, Hakata-ku, Fukuoka 812-0033, Japan; Tel: +81-92-291-3434; Fax: +81-92-291-3424; yokoa@harasanshin.or.jp

List of investigators

Hirokazu Abe, Haruhito Azuma, Shin Egawa, Satoshi Fukasawa, Hiroshi Fukuhara, Ryouhei Hattori, Taro Iguchi, Yukio Kageyama, Sojun Kanamaru, Jun Miki, Takashi Murata, Masayoshi Nagata, Kazuo Nishimura, Norio Nonomura, Eiyu Nozawa, Kenji Numahata, Tetsuo Okuno, Ataru Sazawa, Nobuaki Shimizu, Hiroaki Shimmura, Tatsuya Shimomura, Yutaka Takezawa, Toshiki Tanikawa, Yoshinori Taoka, Hiroji Uemura, Masahiro Uno, Masahiro Yashi, Akira Yokomizo, Junji Yonese

Supplementary Table 1 New antineoplastic therapies initiated during the observation period

	Patients, *n* (%)
Antineoplastic therapy[a]	70 (34.1)
Chemotherapy	22 (31.4)
Hormone therapy	46 (65.7)
Radiopharmaceutical	2 (2.9)

[a]Analysis set: *n* = 205

Supplementary Table 2 Cytotoxic chemotherapies used during the observation period

	Patients, *n* (%)
Cytotoxic chemotherapy[a]	41 (20.0)
Docetaxel	31 (75.6)
Estramustine	10 (24.4)
Cabazitaxel	0
Tegafur/uracil	0
Endoxan	0

[a]Analysis set: $n = 205$

Supplementary Table 3 Treatment duration of enzalutamide by index date

		Treatment duration of enzalutamide								
	Patients, *n* (%)	≤6 months	>6 months to ≤1 year	>1 year to ≤1.5 years	>1.5 years to ≤2 years	>2 years to ≤2.5 years	>2.5 years to ≤3 years	>3 years to ≤3.5 years	>3.5 years	Median (IQR), months
Analysis set	205 (100.0)	45 (22.0)	52 (25.4)	30 (14.6)	25 (12.2)	17 (8.3)	21 (10.2)	12 (5.9)	3 (1.5)	13 (7–24)
Index date										
Nov 2014 to Mar 2015	24 (11.7)	8 (33.3)	3 (12.5)	6 (25.0)	1 (4.2)	1 (4.2)	1 (4.2)	1 (4.2)	3 (12.5)	13 (3–24)
Apr 2015 to Sep 2015	33 (16.1)	5 (15.2)	1 (3.0)	4 (12.1)	2 (6.1)	5 (15.2)	5 (15.2)	11 (33.3)		30 (15–38)
Oct 2015 to Mar 2016	37 (18.0)	7 (18.9)	6 (16.2)	2 (5.4)	4 (10.8)	3 (8.1)	15 (40.5)			23 (9–32)
Apr 2016 to Sep 2016	24 (11.7)	8 (33.3)	2 (8.3)	3 (12.5)	3 (12.5)	8 (33.3)				16 (5–26)

Oct 2016 to Mar 2017	24 (11.7)	3 (12.5)	5 (20.8)	1 (4.2)	15 (62.5)		20 (12–21)
Apr 2017 to Sep 2017	28 (13.7)	6 (21.4)	8 (28.6)	14 (50.0)			12 (7–16)
Oct 2017 to Mar 2018	35 (17.1)	8 (22.9)	27 (77.1)				8 (6–9)

IQR interquartile range

Supplementary Fig. 1 Time to first use of new antineoplastic therapy

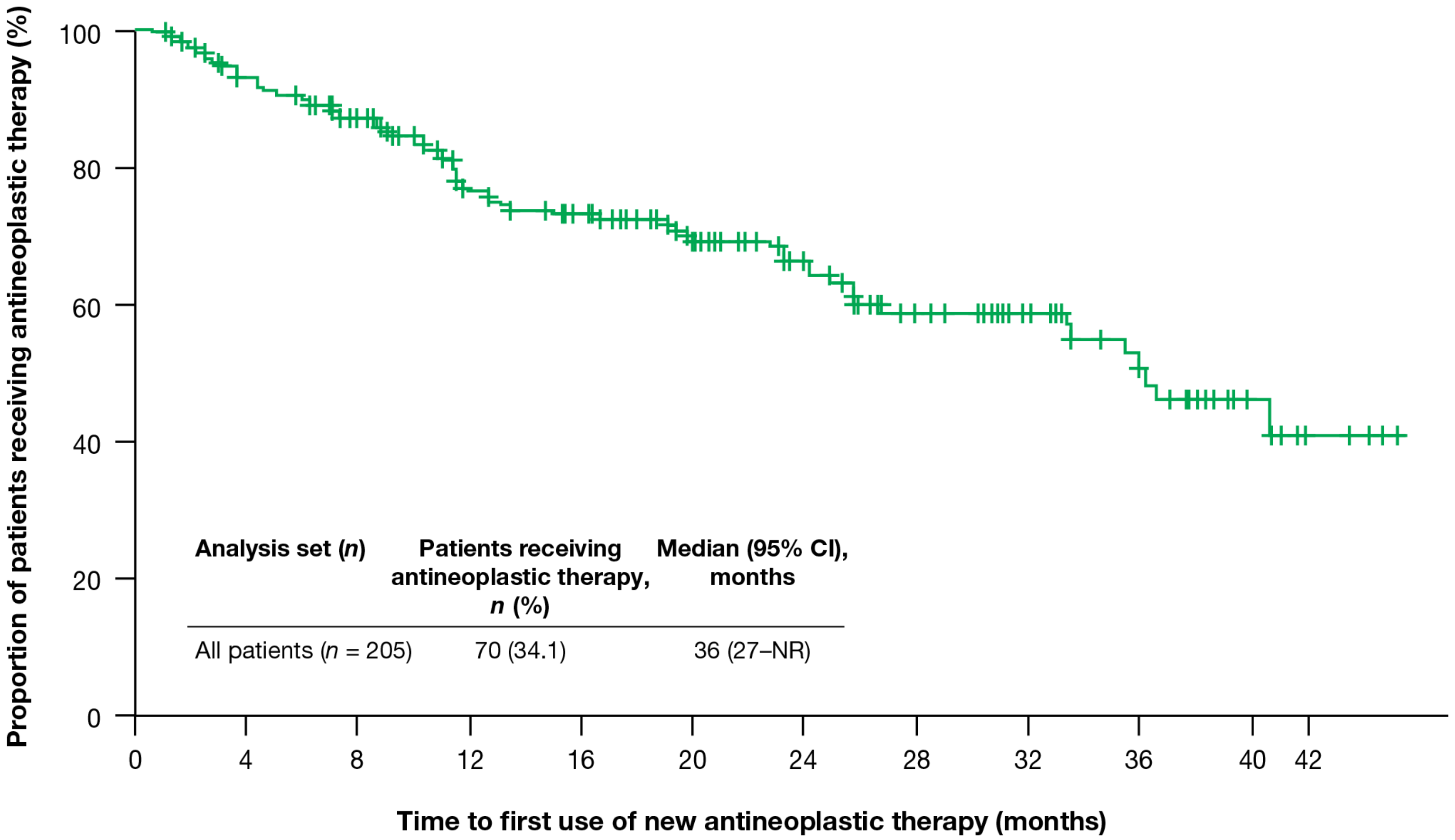

Analysis set (*n*)	Patients receiving antineoplastic therapy, *n* (%)	Median (95% CI), months
All patients (*n* = 205)	70 (34.1)	36 (27–NR)

No. at risk											
All patients	205	183	159	118	105	86	65	45	36	22	9	4

CI confidence interval; *NR* not reached

Supplementary Fig. 2 Time to first use of cytotoxic chemotherapy

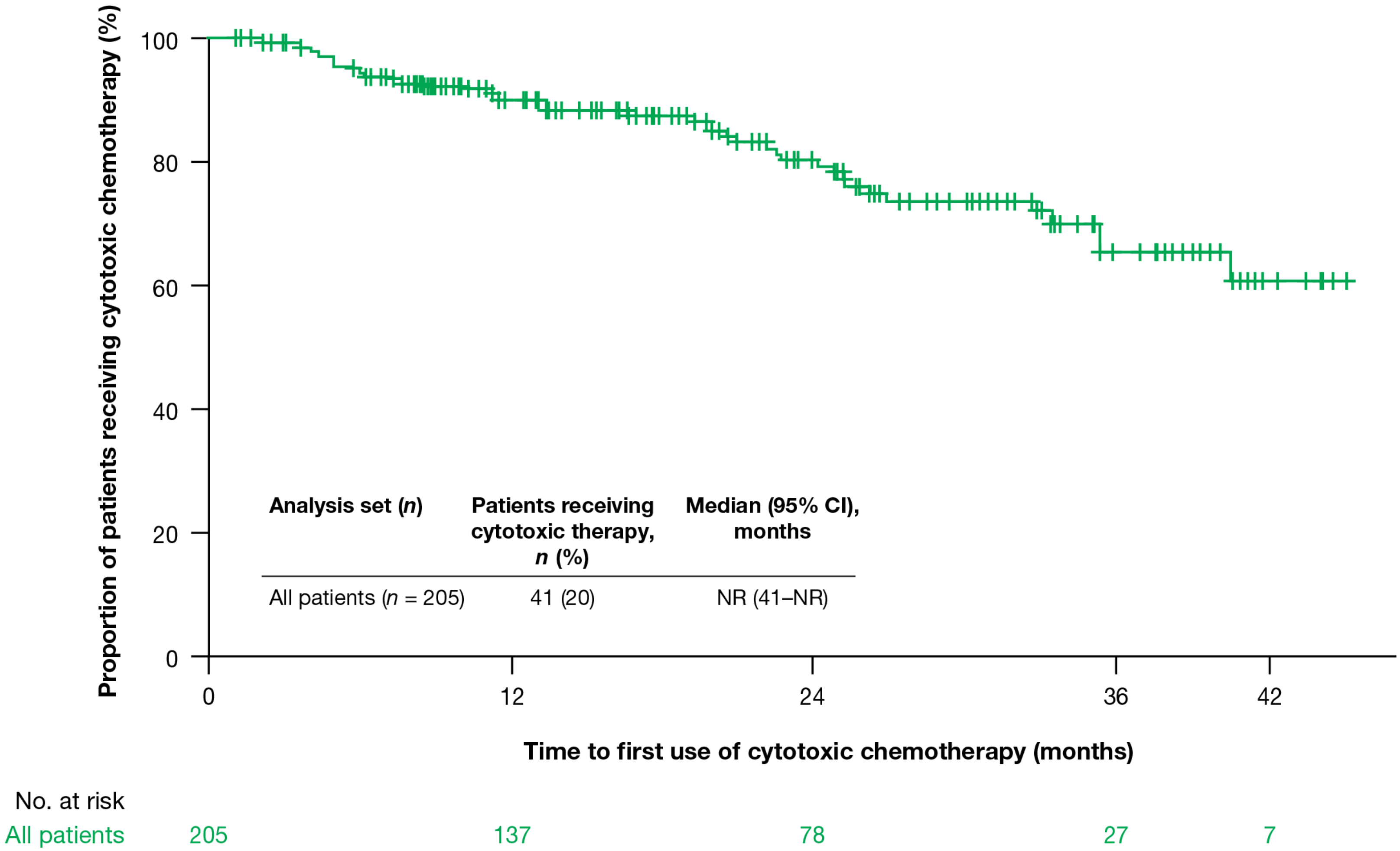

CI confidence interval; *NR* not reached

Supplementary Fig. 3 MFS in (a) all patients and (b) subgroup analysis by median PSA at index date

a

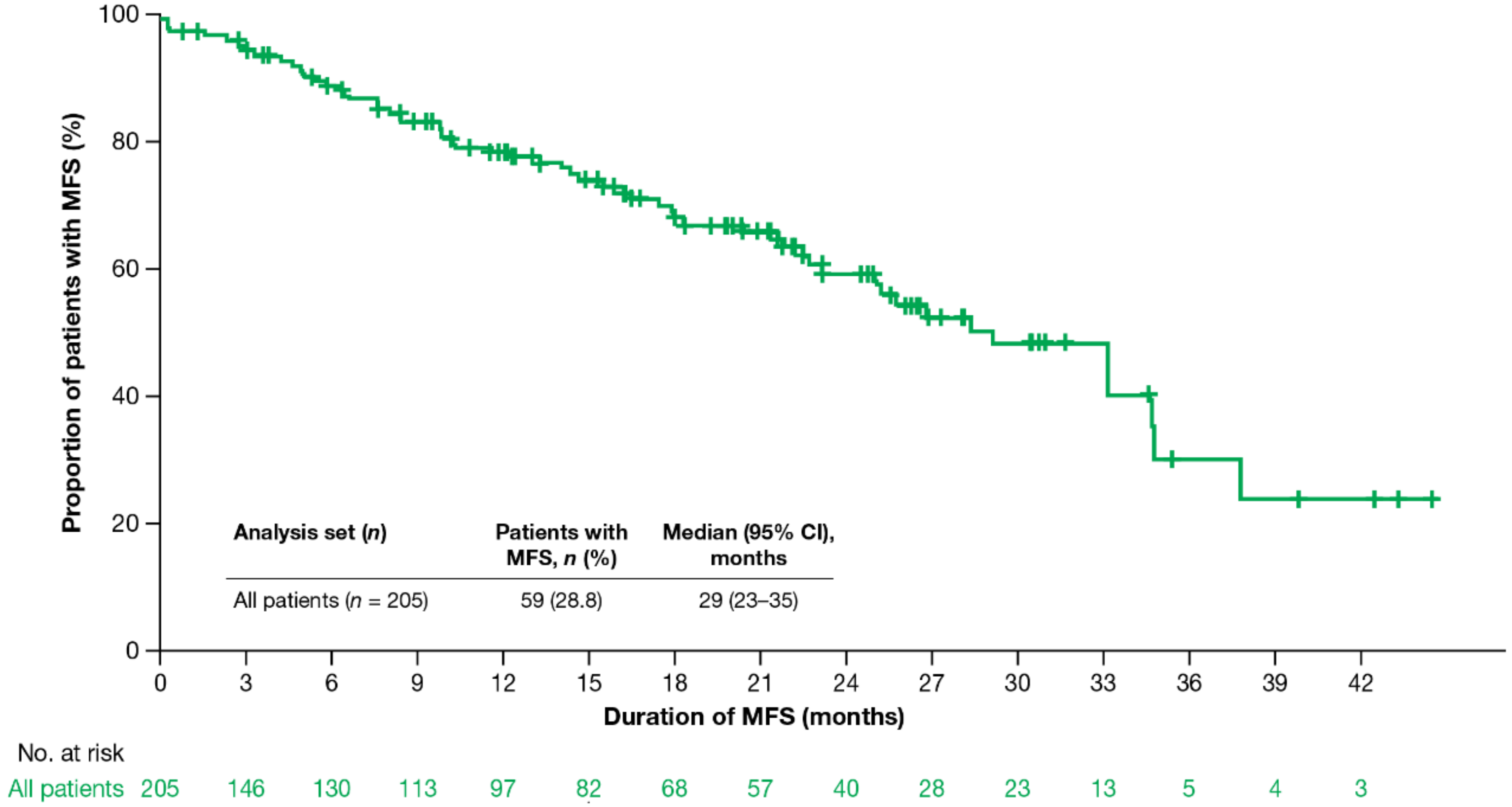

b

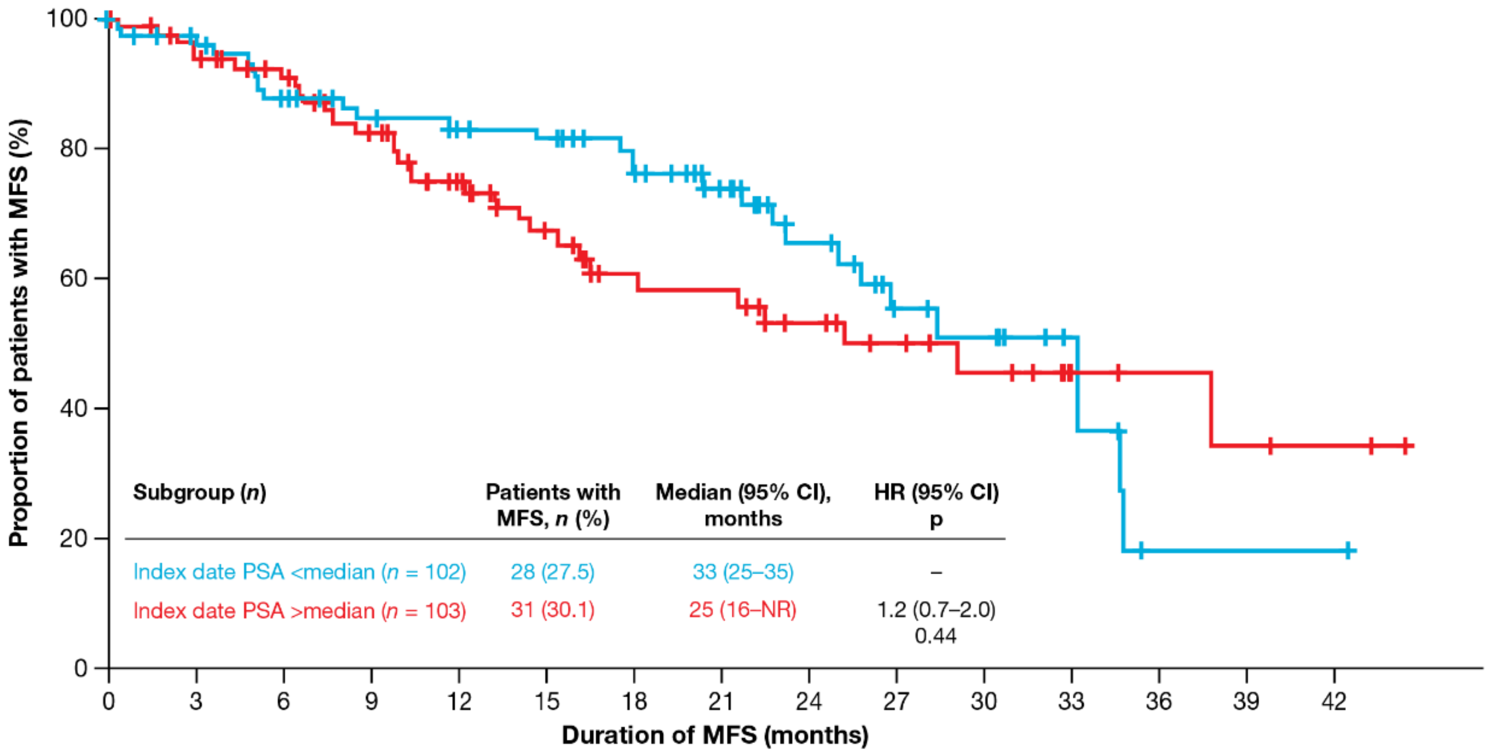

No. at risk															
Index date PSA <median	102	71	63	56	53	49	43	33	22	14	12	7	1	1	1
Index date PSA >median	103	75	67	57	44	33	25	24	18	14	11	6	4	3	2

CI confidence interval; *MFS* metastasis-free survival; *PSA* prostate-specific antigen; *NR* not reached
